# Dietary Trace Minerals

**DOI:** 10.3390/nu11112823

**Published:** 2019-11-19

**Authors:** Elad Tako

**Affiliations:** USDA-ARS, Robert W. Holley Center for Agriculture and Health, Cornell University, Ithaca, NY 14853, USA; elad.tako@ars.usda.gov or et79@cornell.edu

**Keywords:** dietary trace minerals, deficiency, iron, zinc, selenium, copper, vitamin D

## Abstract

Dietary trace minerals are pivotal and hold a key role in numerous metabolic processes. Trace mineral deficiencies (except for iodine, iron, and zinc) do not often develop spontaneously in adults on ordinary diets; infants are more vulnerable because their growth is rapid and intake varies. Trace mineral imbalances can result from hereditary disorders (e.g., hemochromatosis, Wilson disease), kidney dialysis, parenteral nutrition, restrictive diets prescribed for people with inborn errors of metabolism, or various popular diet plans. The Special Issue “Dietary Trace Minerals” comprised 13 peer-reviewed papers on the most recent evidence regarding the dietary intake of trace minerals, as well as their effect toward the prevention and treatment of non-communicable diseases. Original contributions and literature reviews further demonstrated the crucial and central part that dietary trace minerals play in human health and development. This editorial provides a brief and concise overview that addresses and summarizes the content of the *Dietary Trace Minerals* Special Issue.

This monograph, based on a Special Issue of *Nutrients*, contains 13 manuscripts—two reviews and 11 original publications—that reflect the wide spectrum of currently conducted research in the field of dietary trace minerals. The manuscripts in this Special Issue collection include populations from many countries, including the USA, Germany, Australia, Brazil, Poland, Japan, Colombia, Mexico, Saudi Arabia, Russia, Italy, South Korea, and Israel. The presented manuscripts cover a wide variety of topics in the field of dietary trace minerals, with emphasis on the antimicrobial properties of magnesium and the potential to develop healthier food [1], the link between Nrf2 and dietary selenium, iron, zinc, and copper [2], in vivo assessment of fast cooking yellow bean consumption on dietary iron bioavailability [3], the association between nicotianamine and 2′ deoxymugineic acid as enhancers of iron bioavailability in vitro [4], analysis of bioelectrical impedance vector and phase angle on various forms of oral zinc supplementation in children [5], investigation of dietary silicon and its impact on plasma silicon concentrations in human subjects [6], the role of biotin in skin zinc homeostasis [7], the maize germ fraction and its inhibitory effect on iron bioavailability in vitro [8], assessment of the iron bioavailability of iron-biofortified beans in school children [9], investigation of the dietary iron bioavailability of iron biofortified carioca beans in vivo [10], vitamin D supplementation and its effect on serum iron concentrations in adolescents [11], the demonstration of silver ions as a tool for understanding copper metabolism [12], and the dietary and sentinel potential factors that lead to hemochromatosis [13]. This wide spectrum of topics further demonstrates the importance and relevance of dietary trace minerals, as these factors are critical and have a pivotal role in organism (including human) health and physiological functions.

Minerals form only five percent of the typical human diet but are essential for normal health and function. Macrominerals are defined as minerals that are required by adults in amounts greater than 100 mg/day or make up less than one percent of total body weight. Trace elements (or trace minerals) are usually defined as minerals that are required in amounts of 1–100 mg/day by adults or make up less than 0.01 percent of total body weight. Ultra-trace minerals are generally defined as minerals that are required in amounts less than 1 microgram/day [14].

Recommended intakes for trace elements are expressed as Recommended Dietary Allowance (RDA) or Adequate Intake. The Upper Limit is the quantity of the nutrient considered to cause no adverse effects in healthy individuals. These parameters have been estimated for each trace mineral. Previous research demonstrated that: (1) Copper deficiency can be caused by an x-linked mutation of the transport protein mediating copper uptake from the intestine (Menkes disease). It can also be caused by malabsorption after gastrointestinal surgery (including gastric bypass for weight loss and gastric resection for malignancy or peptic ulcer disease), or by ingestion of high doses of zinc. Clinical manifestations include anemia, ataxia, and myeloneuropathy [15]. (2) Iodine deficiency is characterized by goiter and hypothyroidism, which in turn has effects on growth, development, and cognitive function [16]. (3) Selenium deficiency is unusual, but has been reported in parts of China where the local diet is devoid of selenium; this deficiency also occurs in individuals maintained on total parenteral nutrition without trace minerals. Clinical features of selenium deficiency are cardiomyopathy and skeletal muscle dysfunction [17]. (4) Zinc deficiency causes growth retardation in children, hypogonadism, oligospermia, alopecia, dysgeusia (impaired taste), immune dysfunction, night blindness, impaired wound healing, and skin lesions. Infants with an inherited defect in zinc absorption develop a severe deficiency state known as acrodermatitis enteropathica [18,19,20].

The purpose of the current Special Issue is to further expand and add research knowledge on the vital role that dietary trace minerals play in various physiological and metabolic pathways. In addition, it aims to further contribute to knowledge in regards to the relationship between dietary trace minerals’ bioavailability, the microbiome, bioactive compounds, and other metabolic and physiological pathways.

I believe that this Special Issue and collection of manuscripts is a useful summary of progress in various areas related to dietary trace minerals. It also points to additional research needs, including recommendations for future research in the field, in order to better understand the dietary role that trace mineral play and also in regards to specific populations and their dietary requirements, growth and healthy development.
